# Characterizing the tumor microenvironment at the single-cell level reveals a novel immune evasion mechanism in osteosarcoma

**DOI:** 10.1038/s41413-022-00237-6

**Published:** 2023-01-03

**Authors:** Weijian Liu, Hongzhi Hu, Zengwu Shao, Xiao Lv, Zhicai Zhang, Xiangtian Deng, Qingcheng Song, Yong Han, Tao Guo, Liming Xiong, Baichuan Wang, Yingze Zhang

**Affiliations:** 1 Department of Orthopaedics, Union Hospital, Tongji Medical College, Huazhong University of Science and Technology, Wuhan, 430022 China; 2 Department of Orthopaedic Surgery, The Third Hospital of Hebei Medical University, Shijiazhuang, 050051 China; 3 Orthopaedic Institute of Hebei Province, Shijiazhuang, 050051 China; 4 Key Laboratory of Biomechanics of Hebei Province, Shijiazhuang, 050051 China; 5 Animal Center of Hebei Ex & In vivo Biotechnology, Shijiazhuang, 050051 China; 6 Department of Pharmacy, Union Hospital, Tongji Medical College, Huazhong University of Science and Technology, Wuhan, China; 7 Department of Pathology, Union Hospital, Tongji Medical College, Huazhong University of Science and Technology, Wuhan, China

**Keywords:** Bone cancer, Bone cancer

## Abstract

The immune microenvironment extensively participates in tumorigenesis as well as progression in osteosarcoma (OS). However, the landscape and dynamics of immune cells in OS are poorly characterized. By analyzing single-cell RNA sequencing (scRNA-seq) data, which characterize the transcription state at single-cell resolution, we produced an atlas of the immune microenvironment in OS. The results suggested that a cluster of regulatory dendritic cells (DCs) might shape the immunosuppressive microenvironment in OS by recruiting regulatory T cells. We also found that major histocompatibility complex class I (MHC-I) molecules were downregulated in cancer cells. The findings indicated a reduction in tumor immunogenicity in OS, which can be a potential mechanism of tumor immune escape. Of note, CD24 was identified as a novel “don’t eat me” signal that contributed to the immune evasion of OS cells. Altogether, our findings provide insights into the immune landscape of OS, suggesting that myeloid-targeted immunotherapy could be a promising approach to treat OS.

## Introduction

Osteosarcoma (OS), a common primary malignant bone tumor, mainly occurs in children and teenagers.^1^ Advances in surgical technology and neoadjuvant chemotherapy have significantly increased the overall survival rate of OS. Nevertheless, improving the survival rate of recurrent and metastatic diseases still remains a challenge (less than 30% within two years).^2^ Immune checkpoint blockade (ICB) is regarded as a promising therapy for numerous solid tumors, including melanoma, non-small cell lung cancer and kidney cancer.^3^ Recently, immune checkpoint inhibitors that target PD-1 or CTLA-4 have also been tested in OS.^4–6^ However, only a limited number of patients have demonstrated a response to anti-PD-1 immunotherapy in recent clinical trials. Moreover, the impact of anti-CTLA-4 immunotherapy in clinical application for OS remains unclear.^7^

In OS, cancer cells interact with both immune cells and stromal cells to form an immunosuppressive tumor microenvironment (TME), thus enhancing cancer cell immune evasion. The intertumoral heterogeneity is also an important feature of OS, leading to treatment resistance and divergent therapeutic outcomes among patients.^8^ Understanding cancer cell heterogeneity as well as the dynamic tumor immune microenvironment could provide new therapeutic targets to treat OS.

The advent of deep sequencing technology has revolutionized the diagnosis and treatment of diseases. The accumulation of genomic and transcriptomic datasets from large cohorts of clinical samples in TCGA, ICGC and NCBI GEO databases enables researchers to characterize novel therapeutic targets. Conventional bulk RNA sequencing (RNA-seq) is normally performed to determine the mixed gene features of all cellular populations in one sample. Therefore, this method is less likely to detect transcriptional and immunogenic heterogeneity among cell.

The emergence of single-cell RNA-sequencing (scRNA-seq) technology has fundamentally changed the field of tumor biology and provided a strategy to demonstrate TME heterogeneity as well as intercellular communication at the single-cell level.^9,10^ Zhou et al. performed scRNA-seq of 11 patients with OS, and their results revealed the transdifferentiation of malignant cells along with the heterogeneity of tumor-infiltrating T lymphocytes (TILs).^11^ However, the immunoregulatory characteristics of myeloid cells, which might account for most of the tumor-infiltrating immune cells in OS, have not yet been fully investigated.^12^ Myeloid cells, including macrophages, dendritic cells (DCs) and monocytes, play a vital role in tumor immune surveillance through phagocytosis, antigen processing and presentation. Tumor-associated macrophages (TAMs) may also play a critical role in regulating tumor inflammation and angiogenesis to accelerate tumor progression. Given that these myeloid cells can be polarized toward a protumor/antitumor response,^13^ we hypothesize that regulating myeloid cells in the TME can be a promising strategy for OS immunotherapy.

To identify the profile of tumor-infiltrating myeloid cells and the immune heterogeneity of OS, we analyzed published scRNA-seq datasets from the GEO database and bulk RNA-seq data from the Therapeutically Applicable Research to Generate Effective Treatments (TARGET, https://ocg.cancer.gov/programs/target) database to explore the diverse phenotypes and functions of subtypes within the TME. In this study, we identified a tumor-educated “betrayer” DC that suppressed the immune response, deciphered heterogeneous myeloid cells in OS, and predicted the cell‒cell interaction network. Through systematic analyses, our work helps to elucidate the biology of the TME in OS and contributes to the development of immunotherapy in clinical applications.

## Results

### Overview of the osteosarcoma tumor microenvironment at a single-cell resolution

**Fig. 1:**
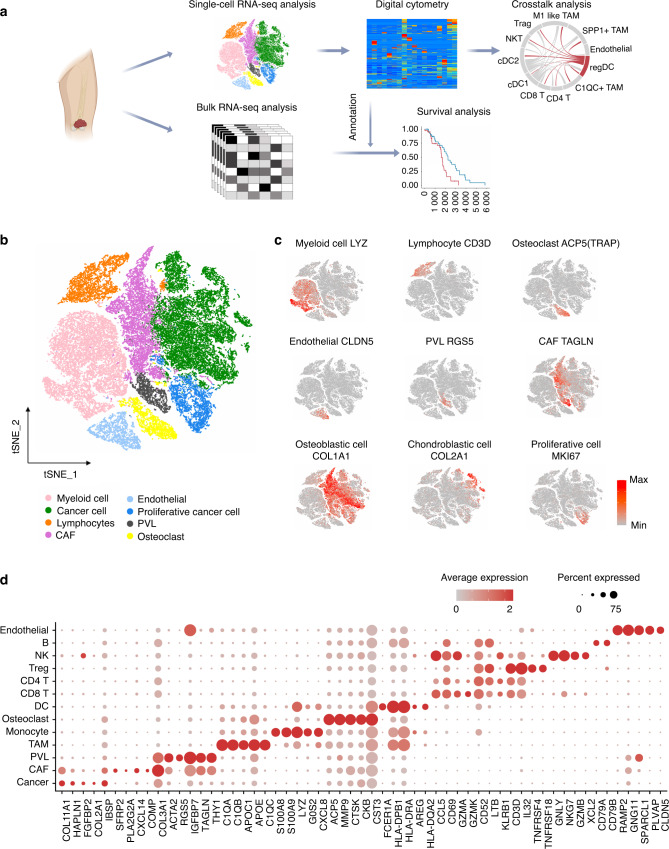
Overview of the osteosarcoma tumor microenvironment at a single-cell resolution. **a** Overall design for investigating the tumor microenvironment in osteosarcoma. **b** T-distributed stochastic neighbor embedding (tSNE) plot of the identified main cell clusters in OS lesions. **c** tSNE plots showing the expression of marker genes of the major cell types detected in this study. Red represents high expression; gray represents low expression. **d** Dot plot showing the specifically expressed genes of the major cell types. Red represents high expression; gray represents low expression. The size of the circle represents the percentage of cells that expressed the indicated genes

In the published single-cell dataset (GSE152048), there are seven primary tumor lesions, two recurrent tumor lesions and two lung metastases. The scRNA-seq data were included in this study and were combined with bulk RNA-seq data of tumor tissue from eighty-five patients (Fig. 1a). After the quality control process (Fig. S1a; b) and removal of the batch effects between patients (Fig. S1c) (see “Materials and Methods”), we identified eight clusters of cells. Briefly, myeloid cells (LYZ^+^), lymphocytes (CD3D^+^), osteoclasts (ACP5^+^), endothelial cells (CLDN5^+^), perivascular-like cells (PVL) (RGS5^+^TAGLN^high^), cancer-associated fibroblasts (CAFs) (TAGLN^low^ACTA2^+^) and proliferative cells (MKI67^+^) were identified in the study (Figs. 1b; c; S2). Among the myeloid cell population, macrophages (APOE^+^CD68^+^), monocytes (S100A8^+^S100A9^+^), and DCs (HLA-DQA1^high^CD14^-^) were annotated. For lymphocytes, we classified CD4 T cells (IL7R^+^), CD8 T cells (CD8A^+^), regulatory T cells (Tregs) (TNFRSF4^+^), natural killer cells (NK) (GNLY^+^GZMB^+^), and B cells (CD79A^+^JCHAIN^+^). Furthermore, PDGFRA^+^CXCL12^+^ CAFs were annotated as inflammatory CAFs (iCAFs) as described by Öhlund D et al.,^14^ while myofibroblast-like CAFs were characterized by high expression of ACTA2 but negative expression of PDGFRA^-^ (Figs. 1d; S2).^15^

### Tumor-associated DCs promote tumor immune tolerance by recruiting Tregs

**Fig. 2:**
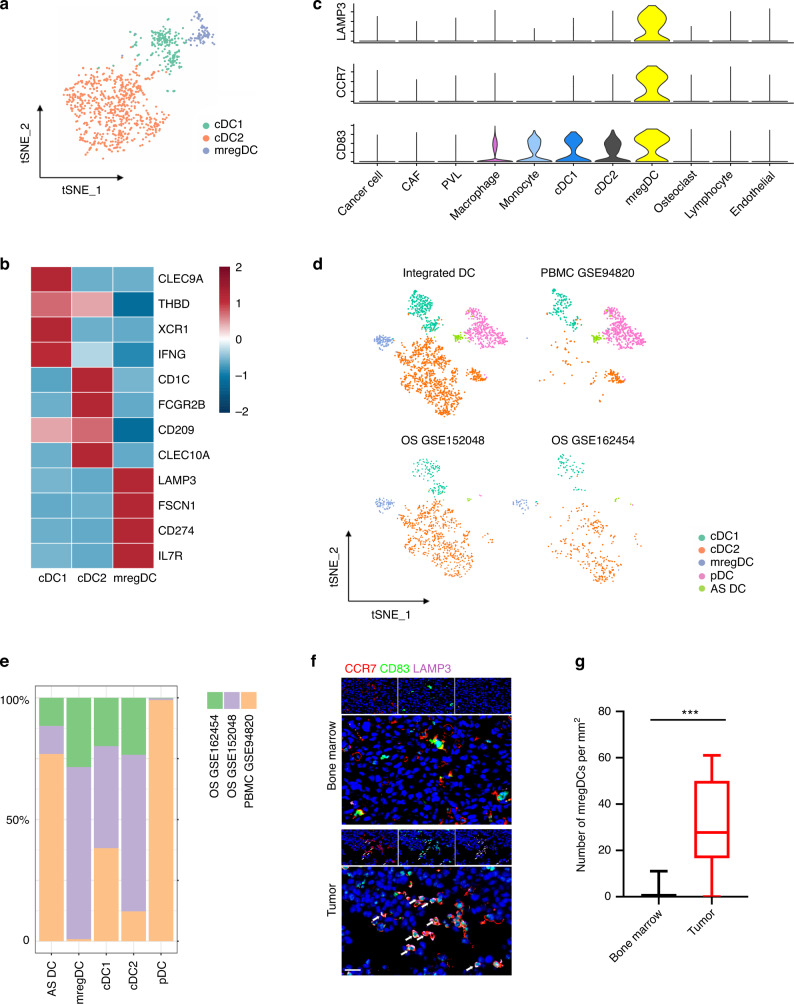
Manifesting mature regulatory dendritic cells (mregDCs) as a tumor-associated DC population. **a** T-distributed stochastic neighbor embedding (tSNE) plot of DCs colored by subgroup clusters. **b** Heatmaps revealing the average gene expression levels of marker genes in each DC cluster. **c** Violin plot showing the specific expression of LAMP3, CD83 and CCR7 in mregDCs. **d** tSNE plots showing the integrated clustering of DCs from OS cohorts and normal peripheral blood mononuclear cells (PBMCs). **e** Cell fractions in the OS cohorts and normal PBMCs. **f** Immunofluorescence staining of mregDC markers in osteosarcoma (OS) primary tumors. The scale bar represents 20 μm. **g** Quantitative analysis of the density of CD83^+^LAMP3^+^CCR7^+^ DCs in the regenerative area from bone marrow and OS tissue sections. Data are the means ± SEMs. ***P < 0.001

Three DC subsets were characterized in OS, including conventional class 1 DCs (cDC1s) (XCR1^+^CLEC9A^+^) and conventional class 2 DCs (cDC2s) (CD1C^+^CLEC10A^+^) (Figs. 2a, b; S3a). In addition, a cluster of CD83^+^CCR7^+^LAMP3^+^ DCs was found in OS and can be referred to as mature regulatory DCs (mregDCs) (Fig. 2a–c).^16^ To investigate whether these mregDCs are tumor specific, we aggregated the DCs from normal peripheral blood mononuclear cells (PBMCs) (GSE94820) and two OS cohorts (GSE152048 and GSE162454) through Harmony packages (Fig. 2d).^17^ We found that mregDCs preferentially existed in the two independent OS cohorts but were nearly absent in normal PBMCs, indicating that mregDCs may be a tumor-associated DC population (Fig. 2d, e). In addition, the number of CD83^+^CCR7^+^LAMP3^+^ DCs was higher in OS than in normal bone marrow, as shown in Fig. 2f, g. These results together suggest the existence of a group of tumor-specific DCs in OS.

**Fig. 3:**
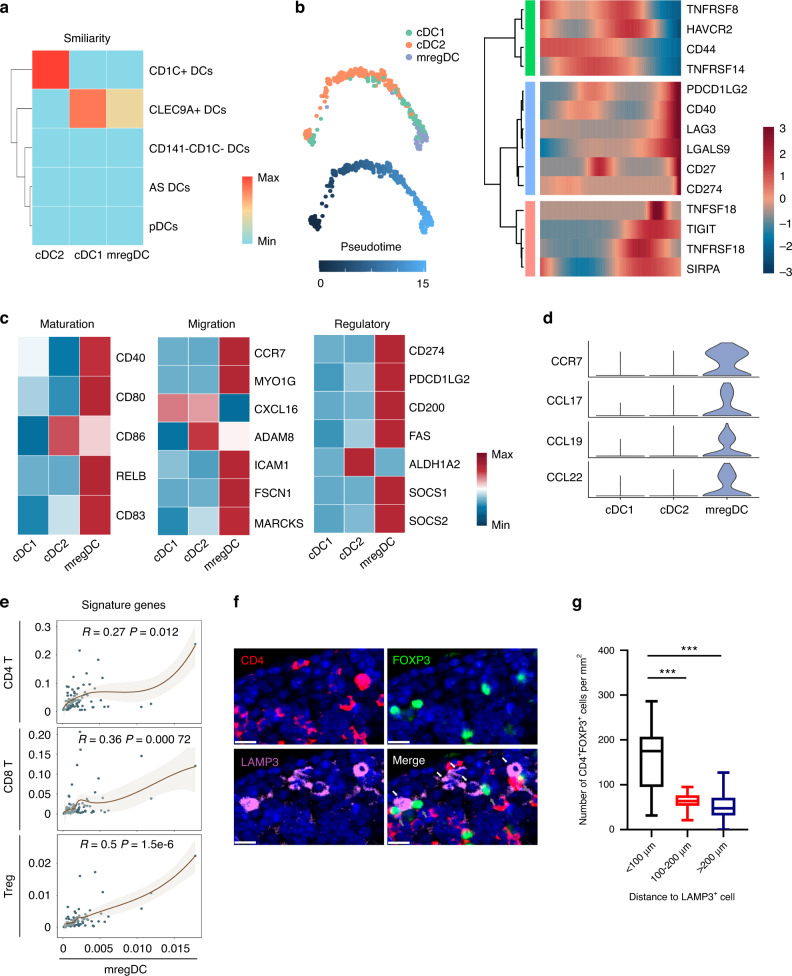
Mature regulatory dendritic cells (mregDCs) promote tumor immune tolerance. **a** Heatmap showing the similarity of dendritic cells from the OS scRNA dataset projected onto different dendritic cells identified from the normal PBMC dataset. Red represents high similarity; blue represents low similarity. **b** Developmental trajectory for DCs inferred by Monocle2 and the heatmap showing the expression changes of coinhibitors and costimulators along the pseudotime trajectory. **c** Heatmaps revealing the average gene expression levels of maturation genes, regulatory markers, and migration genes in dendritic cells. Red represents high expression; blue represents low expression. **d** Violin plots showing the expression of CCR7, CCL17, CCL19 and CCL22 in dendritic cells. **e** Correlation between the mregDC signature and T-cell signatures in the TARGET OS cohort. **f** Representative image of mregDCs and regulatory T cells (Tregs) that colocalized in OS tissues stained by multicolored immunofluorescence staining. White arrows depict mregDCs, while gray arrows indicate Tregs. The scale bar represents 10 μm. g Distribution of CD4^+^FOXP3^+^ cells adjacent or nonadjacent to CD42 mregDCs. Data are the means ± SEMs. *P < 0.05; **P < 0.01; ***P < 0.001

After reclustering of DCs, we used the function TransferData from the Seurat v3 package to calculate the similarity of cells from PBMCs to different subsets identified in the OS dataset. This result suggests similarity between mregDCs and cDC1 subsets (Fig. 3a). To examine the lineage relationship of mregDCs with other DC populations, we performed Monocle2 analysis of DC clusters in OS. The results suggest that mregDCs in OS may originate from cDC1, consistent with previous findings of Cheng et al. and Zhang et al. (Fig. 3b).^18,19^ In addition, the coinhibitors CD274, LAG3, LGALS9, SIRPA, TIGIT, and PDCD1LG2 were upregulated along the pseudotime trajectory (Fig. 3b). Compared with cDC1s and cDC2s, mregDCs exhibited an “activated” phenotype with a higher capacity of migratory ability as well as immune-regulatory ability, indicating that this variable DC subset is mature regulatory DCs (Fig. 3c). More importantly, mregDCs specifically expressed CCR7, CCL17, CCL19 and CCL22, which can recruit multiple types of infiltrating T cells (Fig. 3d).^20,21^ Because the current single-cell RNA-Seq dataset includes only 11 patients, we calculated the correlation between the mregDC signature and T-cell signatures in 85 OS patients from the TARGET website to expand the sample size. The results showed a strong correlation between mregDCs and Tregs (Fig. 3e). Interestingly, staining of tumor sections further confirmed the existence of mregDCs and revealed the physical juxtaposition of mregDCs and Tregs (Fig. 5f). Moreover, the number of Tregs within 100 μm was significantly higher than that in the distant areas (Fig. 3g). To investigate the clinical role of the variable DC subset identified in the present study, we estimated the fraction of every cell type in samples from the TAGET osteosarcoma cohort with CIBERSORTx (Fig. S4a, b).^22^ The score of mregDCs was found to be related to a poorer overall survival rate, and the accumulation of Tregs was correlated with the event-free survival rate (Fig. S3b). In addition, the cell fraction evaluated by CIBERSORTx suggested a positive correlation between mregDCs and Tregs (Fig. S3c). These results suggest the possibility that mregDCs promote tumor immune tolerance through recruitment of Tregs in the OS TME.

### The heterogeneity of cancer cell immunogenicity

**Fig. 4:**
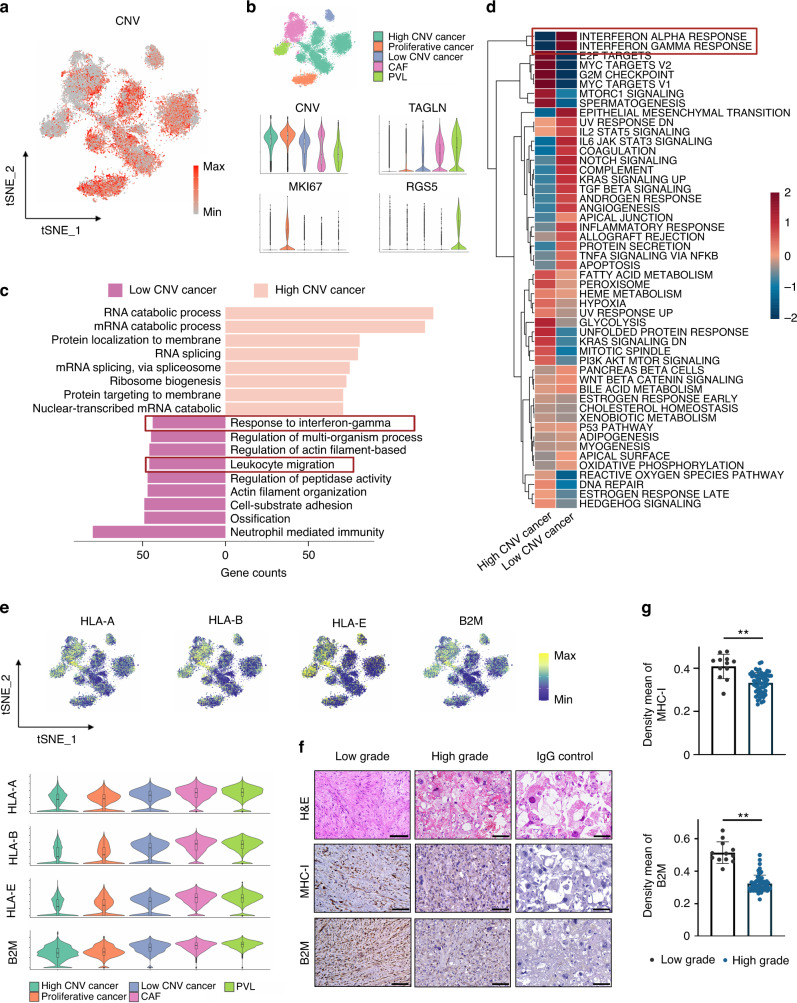
The heterogeneity of cancer cell immunogenicity. **a** tSNE plot representing the single-cell copy number variations (CNVs) of cancer cells. Red represents high levels of CNV, and gray represents low levels of CNV. **b** tSNE plots of stromal cells colored by cluster (top); violin plots of CNV score and marker gene expression levels (bottom). **c** Bar plot showing the enriched Gene Ontology enrichment of differentially expressed genes between CNV high and low cancer cells. **d** Heatmap revealing the differentially regulated pathways between CNV high and low cancer cells scored by GSVA. Red represents high scores; blue represents low scores. **e** tSNE plots and violin plots showing the expression of HLA-A, HLA-B, HLA-E and B2M in stromal cells. **f** and **g** Representative immunohistochemistry and mean density of MHC-I and B2M expression in low-grade and high-grade OS tissue. Scale bar, 100 μm. Data are the means ± SEMs. *P < 0.05; **P < 0.01

Copy number variations (CNVs) have been shown to be an effective strategy to identify more aggressive clones of cancer cells. Zhou et al. revealed that more canonical CNVs accumulated in chondroblastic OS lesions, suggesting that chondroblastic cancer cells are a less differentiated OS type.^11^ However, whether high CNV leads to immune escape remains unclear. Thus, we examined the relationship between CNV and the immune response in OS in the current study. We integrated the stromal cells (Fig. S5a) and estimated the CNV of each cell by the inferCNV package (Figs. 4a, b; S5b). The results revealed that cancer cells accumulated a larger number of CNVs than fibroblast cells. Interestingly, we observed a cluster of low CNV cancer cells in the stromal cells. PySCENIC^23^ was applied to perform transcription factor and motif analysis. Transcription factor (TF) motifs, including CEBPB (+), FOSB (+), SAP30 (+) and ATF4 (+), were significantly upregulated in CNV high cancer cells, while IRF3 (+), ETV7 (+), STAT1 (+) and IRF7 (+) were downregulated (Fig. S5c), indicating promising new regulatory networks driven by TFs in OS cells. In addition, both Gene Ontology (GO) enrichment analysis^24^ and gene set variation analysis (GSVA)^25^ of MSigDB hallmark gene sets revealed that the interferon-gamma response was relatively enriched in CNV low cancer cells (Fig. 4c, d). As transcriptional downregulation of MHC-I is one of the most important factors that impairs the antitumor effect of IFN-γ signaling,^26^ we subsequently examined the expression of MHC-I molecules at the mRNA level. We found that cancer cells with higher levels of CNV displayed lower levels of MHC-I genes (HLA-A, HLA-B and HLA-E) and the B2M gene, suggesting that these cancer cells were less immunogenic (Fig. 4e). To further examine whether the downregulation of MHC-I can be generalized across osteosarcoma, we evaluated the expression of MHC-I and B2M through immunohistochemistry (IHC) staining in sections from OS patients. The results showed that high-grade OS downregulated the expression of MHC-I and B2M (Fig. 4f, g). Based on these findings, we believe that the downregulation of the interferon signaling pathway and MHC class I molecules in high-grade OS may lead to immune evasion.

### CD24 signaling regulates the macrophage-mediated immune response to OS

Accumulating evidence suggests that the TME polarizes macrophages toward a protumor phenotype in multiple types of cancers.^27^ In the current study, the results of the analysis of macrophage subsets revealed robust expression of MRC1 (encoding CD206) and CD163 (Fig. S6a–c). In addition, MHC-II genes were downregulated in C1QC^+^ TAMs and SPP1^+^ TAMs (Fig. S6d). These results indicate the existence of an M2-like phenotype in most macrophages in OS. However, the underlying mechanisms that drive immune tolerance are not yet fully understood.

**Fig. 5:**
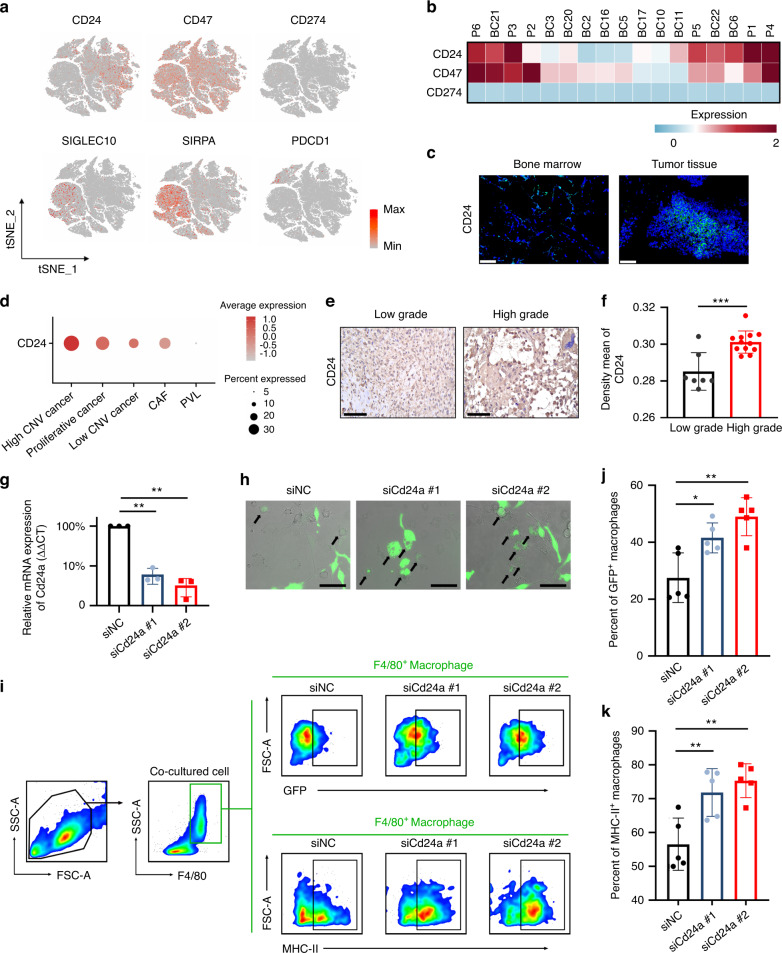
CD24 protects OS cells from macrophage attack. **a** Expression of the immune checkpoints CD24, CD47, and CD274 and their receptors overlaid onto tSNE plots. Red represents high expression; gray represents low expression. **b** Heatmap showing the normalized expression of CD24, CD47, and CD274 in each patient. Red represents high expression; blue represents low expression. **c** Fluorescence in situ hybridization (FISH) imaging of CD24 in tumor tissues and bone marrow from the patients. The scale bar represents 100 μm. **d** Dot plots showing the expression of CD24 in stromal cells. Red represents high expression; gray represents low expression. The size of the circle represents the percentage of cells that expressed the indicated genes. **e** and **f** Representative immunohistochemistry and mean density of CD24 expression in low-grade and high-grade OS tissue. Scale bar, 100 μm. **g** Relative expression of Cd24a in K7M2 cell lines that were transfected with Cd24a siRNA or scramble siRNA. Scale bar, 100 μm. Data are the means ± SEMs. *P < 0.05; **P < 0.01. **h** Representative live fluorescence microscopy images of in vitro phagocytosis of K7M2 cells (GFP^+^ green) by BMDMs in the presence of Cd24a siRNA or scramble siRNA. **i-k** Representative images of the gating strategy and statistical analysis for the in vitro phagocytosis assay and the M1-like macrophage assay. Data are the means ± SEMs. *P < 0.05; **P < 0.01

Cancer cells were reported to evade clearance by immune cells through the overexpression of antiphagocytic surface proteins, including CD47 and programmed cell death ligand 1 (encoded by CD274). CD24 is a novel “don’t eat me” signal that inhibits Toll-like-receptor-mediated inflammation and cellular engulfment by macrophages.^28^ The expression of CD24 and CD47 was stronger than that of CD274, indicating a role of macrophage-mediated immune escape rather than T-cell-mediated immune evasion in OS (Fig. 5a, b). At the single-cell resolution, we found that CD47 was highly expressed by almost all cell types, while CD24 was preferentially expressed by OS cells (Fig. 5a). CD24 exhibited higher mRNA expression in OS tissues than in normal bone marrow from the same patients, as shown by fluorescence in situ hybridization (FISH) (Fig. 5c). Moreover, high CNV cancer cells were found to express higher CD24 than low CNV cancer cells and fibroblasts (Fig. 5d). IHC staining also demonstrated stronger expression of CD24 in high-grade OS (Fig. 5e, f). These results together illustrate that CD24 is a potential immunotherapy target in OS.

**Fig. 6:**
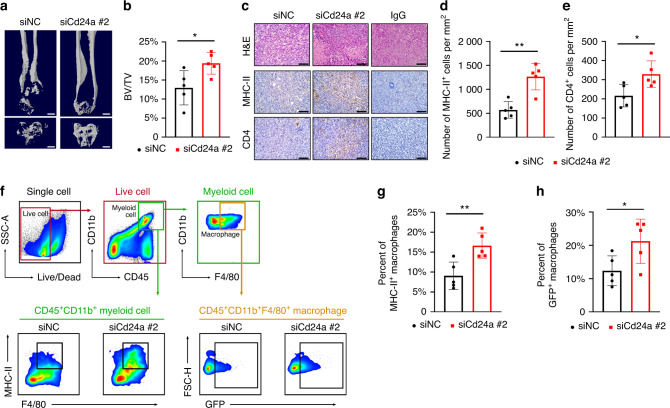
Interference with the expression of Cd24a promotes phagocytosis and the M1-like phenotype of macrophages in vivo. **a**, **b** Representative images and analysis of micro-CT scanning of the distal femur in each group. Scale bar, 1 mm. BV, bone volume; TV, total volume. **c**–**e** Representative images and analysis of IHC staining of MHC-II and CD4 in tumors treated with cholesterol-modified Cd24a siRNA or scramble siRNA. Scale bar, 100 μm. **f–h** Representative images of the gating strategy and statistical analysis for the in vivo phagocytosis assay and M1-like macrophage assay. Data are the means ± SEMs. *P < 0.05; **P < 0.01; ***P < 0.001

To investigate the role of CD24 in regulating the macrophage-mediated immune response in OS, we treated bone marrow-derived macrophages (BMDMs) with IL-4 to generate M2-like macrophages and cocultured these less phagocytic macrophages with the GFP^+^ K7M2 osteosarcoma cell line for 36 h. We found that interference with Cd24a in the K7M2 cell line potentiates phagocytosis as measured by live-cell microscopy (Fig. 5g, h). Similarly, fluorescence-activated cell sorting (FACS)-based measurements revealed a significant increase in phagocytosis upon transfection with Cd24a siRNA compared to the scramble siRNA (Fig. 5i, j). In addition, the BMDMs cocultured with Cd24a knockdown cancer cells had a more inflammatory phenotype (Fig. 5i, k). To investigate whether the protection of phagocytosis conferred by downregulating CD24 could be recapitulated in vivo, we treated mice bearing periosteal osteosarcoma with cholesterol-modified Cd24a siRNA or scramble siRNA at a dose of 1 OD every two days through intratumor injection. Three weeks after engraftment, we observed significantly reduced tumor tumorigenicity in the Cd24a siRNA group compared to the scramble siRNA group as measured by micro-CT scanning (Fig. 6a, b). Moreover, robustly increased MHC-II^+^ cells and infiltrating CD4 T cells were observed in the Cd24a siRNA group, as shown by IHC staining (Fig. 6c–e). FACS analysis also revealed increases in phagocytosis as well as antigen presentation phenotype (Fig. 6f–h). In summary, our results revealed that OS cells evade the macrophage-mediated immune response through CD24 signaling.

### Cell‒cell interactions within OS

**Fig. 7:**
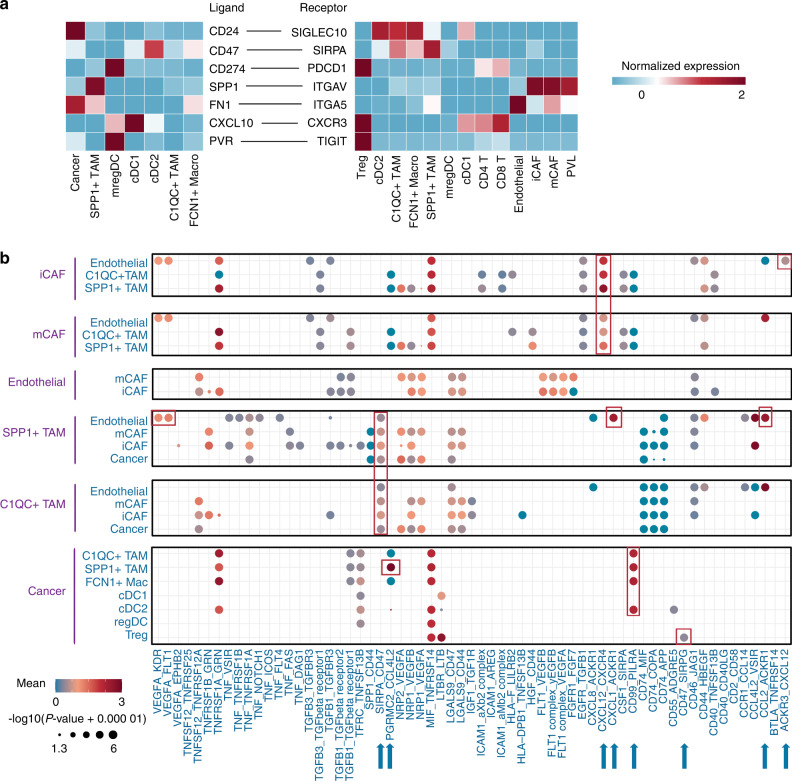
Predicted cell–cell interaction network in the OS TME. **a** Heatmap showing the normalized expression of ligands and receptors in the indicated clusters in OS. Red represents high expression; blue represents low expression. **b** Bubble plots showing ligand‒receptor pairs predicted between cell clusters. Dot size indicates the *P* value generated by CellPhoneDB, colored by mean attraction strength levels

Given that exploring the cell‒cell interaction LR pairs can provide new information about the OS TME, we calculated the attraction strengths of ligand‒receptor pairs in the scRNA-seq dataset. The cell‒cell interactions in OS were also interrogated by CellPhoneDB.^29^ The enrichment of the CD24-SIGLEC10 LR pair between cancer cells and multiple macrophage subsets revealed the immunoregulatory role of CD24 in OS, as we described above. In addition, the robust expression of SPP1 and ITGAV in SPP1^+^ TAMs and CAFs suggested that SPP1^+^ TAMs may promote directional cancer cell migration by aligning fibronectin in CAFs (Fig. 7a).^30,31^ Next, we focused on the heterogenetic cell‒cell interactions of DC subsets. We found that cDC1 had a strong ability to induce multiple T-cell infiltration through CXCL10-CXCR3, whereas mregDCs showed the highest immunosuppression potency through CD274-PDCD1 and PVR-TIGHT interactions with Tregs (Fig. 7a). In addition, we found that CAFs and SPP1^+^ TAMs interacted with endothelial cells through the ACKR3-CXCL12 and CCL2/CXCL1-ACKR1 axes (Fig. 7b). Our analyses suggested a role for the ACKR family in angiogenesis in OS. Other ligand‒receptor pairs involved interactions between cancer cells and SPP1^+^ TAMs through PGRMC2-CCL4L2 and interactions between CAFs and TAMs through CXCL12-CXCR4. Of note, the OS samples were composed of osteoblastic osteosarcoma and chondroblastic osteosarcoma from primary, recurrent, and metastatic lesions. In these cases, cells cannot communicate with each other, as they do not reside in the same TME. Thus, we separately predicted cell‒cell interactions in different lesions. These results revealed distinct cell‒cell interaction modes in various OS types, which may require precise personalized treatments. Overall, our analysis of scRNA-seq data suggests a role of myeloid cells in the TME through interacting with immune and stromal cells in OS (Fig. S8).

## Discussion

Currently, the treatment of advanced OS is still very challenging. In the present study, we leveraged the advantages of scRNA-seq technology and bulk RNA-seq to explore the immune heterogeneity of cancer cells as well as the atlas of myeloid cells within OS patients. An immunoregulatory subset of DCs was identified in OS tissue. The classification of the malignant cells into CNV high or low groups revealed diverse immunogenicity in OS. In addition, CD24 was characterized as a novel “don’t eat me” signal that mediated the immune escape of OS cells.

Dendritic cells, the most efficient professional antigen presentation cells (APCs), are critical in T-cell priming, activation, and differentiation. Based on their cell surface markers, human dendritic cells can be classified into conventional class 1 DCs (cDC1s), conventional class 2 DCs (cDC2s), and plasmacytoid DCs. Recently, the existence of a new type of dendritic cell (cDC3) in mice and humans has been proven by single-cell sequencing.^32^ cDC1 is essential for CD8 T-cell priming and activation, which is important for antitumor and antiviral immunity, while cDC2 is involved in CD4 T-cell responses.^33,34^ Moreover, recent studies found that cDC1s were not only essential for priming CD8 T cells but were also required for the licensing of CD4 T cells in tumors.^35^ In the current study, CXCL10-CXCR3 was enriched between cDC1 and multiple T cells (Fig. 7a), indicating the role of cDC1 in the recruitment or activation of T cells. In addition, cDC1s were the only tumor-infiltrating immune cells that were associated with better prognosis in the current study (Fig. S3b). These results suggest the core role of cDC1s in immune priming as well as the necessity to develop cDC1 recruitment strategies.^36^

Although mature immunoregulatory DCs have been identified in various cancers, the presence of this subgroup in osteosarcoma has not yet been confirmed.^16,19^ The mregDCs (CCR7^+^LAMP3^+^CD83^+^) in the current study can be referred to as CCR7^+^ DCs that were found by Zhou et al.^11^ As they mainly focused on the heterogeneity of cancer cells and T cells, the role and function of this DC subset were not fully annotated. In this study, the interaction between mregDCs and Tregs was identified through CD274-PDCD1 and PVR-TIGIT signaling, as well as their physical juxtaposition. Given that mregDCs were enriched in OS but were absent in PBMCs, we speculate that mregDCs are tumor-associated DCs that negate antitumor immunity in OS.^19,37^ Further studies are still needed to better understand the regulation and differentiation of mregDCs. The marker genes of the DC populations identified in this study can also be used to characterize this DC subset for further studies.

Many cancers evade immune surveillance by suppressing the expression of major histocompatibility class I (MHC-I).^38^ Loss of MHC-I expression enables tumor cells to escape killing by cytotoxic T lymphocytes.^39^ Moreover, transcriptional repression of MHC I has been reported to be associated with resistance to cancer immunotherapy.^40^ In our study, the transcription of MHC I and the interferon-gamma response were repressed in high CNV cancer cells (Fig. 2b). The results suggested that downregulating MHC-I on tumor cells may be a driving mechanism of immune evasion in OS. Moreover, emerging evidence suggests a role for antiphagocytic signals in immune evasion. Through the expression of “don’t eat me” signals, tumors are capable of escaping macrophage-mediated phagocytosis. CD47 is a classical “don’t eat me” signal that binds to its receptor SIPRα on macrophages to protect cells from phagocytosis.^41^ Mohanty et al. reported an additive therapeutic effect of CD47 mAb in animal models of OS.^42,43^ However, CD47 is also expressed in normal immune cells and erythrocytes. As a result, anemia and neutrophil count decreases were frequently observed in patients who underwent CD47 blockade therapy.^44^ To address this issue, we tried to explore novel macrophage-targeted immune therapies. CD24 is a cancer stem cell marker that is critical for the maintenance, self-renewal, and differentiation of OS.^45–47^ Recently, CD24 was identified as a novel “don’t eat me” signal through the CD24-Siglec10 interaction in cancers.^48^ However, scant attention has been given to the role of CD24 in innate immune evasion in OS. Our results showed that CD24 was a tumor-specific “don’t eat me” signal in OS. We also found that high-grade OS cells exhibited robust expression of CD24. More importantly, interference with the expression of CD24 potentiated phagocytosis and activation of macrophages in OS. These results could serve as evidence in support of the therapeutic potential of CD24 blockade in OS immunotherapy.

In summary, we constructed a single-cell atlas of osteosarcoma cells and myeloid cells across scRNA-seq data and bulk RNA-seq data, with the aim of verifying the role of mregDCs in OS. Our study also revealed CD24 as a novel “don’t eat me” signal in OS, suggesting new avenues for potential therapeutic treatments of OS.

## Materials and methods

### Data acquisition

The scRNA-seq of OS has been described by Zhou et al. and Liu et al.^11,49^ The processed count matrix was directly obtained from GSE152048 and GSE162454, and the clinical data of these patients were obtained from their supplementary data. The clinical sample bulk RNA-seq data were acquired from Therapeutically Applicable Research to Generate Effective Treatments (TARGET, https://ocg.cancer.gov/programs/target). The reference scRNA-seq data of the DC population were obtained from GSE94820.

### Analysis of scRNA-seq data

Data processing of scRNA-seq data was mainly performed by Seurat (version 3.0.1).^50^ Briefly, low-quality single cells were eliminated through a set threshold with the number of UMIs, features and mitochondrion-derived genes. The intergradation of data from the patients was performed by the IntegrateData function in Seurat to remove the batch effects among patients, and the top 3 000 variable genes were used to calculate intergradation anchors in this process. Subsequently, the NormalizeData function in Seurat was used to normalize the data matrix. The unsupervised clustering of the main cell subtypes was performed by the FindClusters function in Seurat and visualized with 2D UMAP or t-distributed stochastic neighbor embedding (tSNE). Then, the markers of each cell cluster were identified by the FindAllMarkers function in Seurat for annotation. Monocle2 was used to decipher the transcriptional trajectories of macrophages.

### Patients and tumor samples

All clinical samples were obtained from the Department of Orthopedics, Union Hospital of Tongji Medical College, Huazhong University of Science and Technology, Wuhan, China. The murine osteosarcoma K7M2 cell line was purchased from Zhongqiaoxinzhou Biotechnology Co., Ltd. (Shanghai, China). A tumor-bearing mouse model was established by inoculating 2 × 10^6^ K7M2 cells with Matrigel matrix (Sigma-Aldrich E1270) into the right flank of 6-week-old female Balb/c mice. Then, these subcutaneous tumors were cut into 1 × 1 × 1 mm^3^ lumps and transplanted onto the periosteum of the distal femur in 6-week-old female Balb/c mice. All experimental processes were approved by the Institutional Review Board of Union Hospital, Tongji Medical College, Huazhong University of Science and Technology and Ethics Committee of Hebei Ex & In Vivo Animal Center.

### Cell culturing and in vitro phagocytosis assay

The GFP-K7M2 cell line was obtained from Qijing Biological Technology Co., Ltd. (Wuhan, China). Bone marrow-derived macrophages (BMDMs) were obtained by culturing bone marrow from 6-week-old Balb/c mice with DMEM containing 10% FBS and 20 ng·mL^−1^ m-CSF (R&D, 416-M-050). On Day 5 of culture, the medium was refreshed again, and 20 ng·mL^−1^ IL-4 was added for 4 days to induce M2-like BMDMs. BMDMs were cocultured with GFP-K7M2 cells in phagocytosis assay wells to observe the phagocytosis rate. All phagocytosis assay wells were stained with anti–mouse F4/80–Super Bright 645 (eBioscience, 64-4801-82; 1:200) for 30 min prior to flow cytometry analysis.

### siRNA transfection and in vivo treatment

siRNA transfection was performed using an RNATransMate kit (Sangon Biotech, E607402) at 20 nmol·L^−1^ for 12 h. The knockdown efficiency was validated with qRT‒PCR. For in vivo RNA interference, cholesterol-modified Cd24a siRNA or scramble siRNA were designed and synthesized by Sangon Biotech Co., Ltd., (Shanghai, China) and were intratumorally injected at a dose of 1 OD every second day. The sequence of siRNA we used can be found in Table S1.

### Fluorescence in situ hybridization imaging

For fluorescence in situ hybridization (FISH), the bone marrow was centrifuged before fixation, while tissues were fixed directly in 4% PFA/PBS overnight. These tissues were dehydrated before embedding. For all FISH imaging, sections were washed with proteinase K (Servicebio, G1205) before preliminary hybridization. Then, we removed the prehybridization solution, added probe hybridization solution at a concentration of 500 nmol·L^−1^, and hybridized overnight at 42 °C. Finally, the sections were incubated with DAPI for 8 min in the dark. The imaging was collected by Pannoramic MIDI II-3Dhistech and analyzed by CaseViewer Software (version 2.4). The sequence of the probe we used can be found in Table S1.

### CNV inference of cancer cells

The InferCNV package (version 1.2.2; https://github.com/broadinstitute/inferCNV/wiki) in R was applied to infer the CNVs in OS cells. We identified and annotated endothelial cells and fibroblast cells based on the expression of known marker genes and then introduced them as a reference for CNV estimation.

### Pathway analysis

DEGs between clusters were identified by the FindMarkers function of Seurat with the cut off threshold at adj. *P* val <0.01 and fold change (FC) > 1.3. The DEGs were subsequently used for GO enrichment analysis as well as KEGG analysis with clusterProfiler. The GSVA package in R was applied for GSVA.

### Gene regulatory network analysis

SCENIC is an algorithm to identify transcription factors and cell states through the analysis of gene regulatory networks from scRNA-seq data. The pySCENIC refactored and reimplemented this algorithm in Python. The area under the curve (AUC) of each regulon of cells was calculated by pySCENIC. The difference in AUCs among cell clusters was identified through the Limma package, and the regulons with an adjusted p value (adj. p val) less than 0.05 were used for further analysis.^51^ The AUCell package in R was applied to embed the AUC score into UMAP.

### Immunofluorescence

Whole tumors were fixed in 4% PFA/PBS overnight and dehydrated before embedding. For all morphologic examinations, 4 μm-thick sections were prepared for H&E and IHC staining. H&E and IHC staining were performed according to the manufacturer’s protocols (Solarbio, G1120). For multi-immunofluorescence imaging of tumor tissues, sections were first stained with primary antibodies in PBS. After staining with horseradish peroxidase-conjugated secondary antibodies, the sections were incubated with fluorescent tyramide signal amplification (TSA) reagent. Then, the antigen-antibody complexes on sections were eluted. Subsequent immunofluorescence staining was performed. Multispectral images were collected by Pannoramic MIDI II-3Dhistech and analyzed by CaseViewer Software (version 2.4).

The following reagents were used in IHC staining: anti-MHC-I (rabbit, 1:100, HuaAn, ET1702-47), anti-B2M (rabbit, 1:100, ABclonal, A12404), anti-MHC-II (rabbit, 1:200, Invitrogen, PA5-116876), anti-CD4 (rat, 1:50, BD Pharmingen, 550278), anti-CD24 (rabbit, 1:100, ABclonal, A2207), anti-FOXP3 (rabbit, 1:1 000, Servicebio, GB112323), anti-CD63 (mouse, 1:100, Arigo, ARG41312), anti-CCR7 (rabbit, 1:100, HUABIO, ET1602-22), anti-CD83 (rabbit, 1:1 000, ABclonal, A2040), HRP conjugated Goat Anti-Rabbit IgG (1:500, Servicebio, GB23303), CY3-Tyramide (1:2 000, Servicebio, G1223), FITC-Tyramide (1:1 000, Servicebio, G1222), CY3-Tyramide (1:2 000, Servicebio, G1223), and Cy5 conjugated Goat Anti-Mouse IgG (1:400, Servicebio, GB27301).

### Flow cytometry assay

The tumors were minced and digested with digestion cocktail (collagenase 1.5 mg·mL^−1^, hyaluronidase 1.5 mg·mL^−1^, DNase 20 μg·mL^−1^) at 37 °C for 30 min. Then, the suspension was transferred onto a 70-μm cell strainer to remove undigested tissue. ACK lysis buffer was used to exclude red blood cells. After CD16/32 blocking (BD Biosciences, 553141, 1:100) for 30 min, the suspensions were incubated with antibodies for 30 min at 4 °C. The antibodies used in the experiments were as follows: Fixable Viability Stain 510 (BD Biosciences, 564406, 1:1 000), anti–mouse CD45–APC–Cy7 (BD Biosciences, 557659; 1:100), anti-mouse CD11b–BB700 (BD Biosciences, 746004; 1:100), anti–mouse F4/80–Super Bright 645 (eBioscience, 64-4801-82; 1:200), and anti–mouse MHC-II–APC (BD Biosciences, 562823; 1:100). Cells were resuspended in staining buffer before flow cytometry and were analyzed on a BD FACSCelesta™ flow cytometer. Data were analyzed using FlowJo version 10.0 (Treestar).

### Micro-CT scanning (μCT) analyses

The tumor-bearing leg was harvested, and the normal soft tissue around the femur was removed. After that, μCT analyses were performed with a SkyScan 1174 μCT scanner. The scanning procedure was performed at 63 kV with a 153-μA current and a resolution of 9 μm/pixel. CTAn (version 1.9, SkyScan) was used for quantitative analysis as well as 3D reconstruction. CTVol (version 2.0, SkyScan) was used for the visualization of 3D models.

### Cell–cell interaction analysis

CellPhoneDB is a Python-based algorithm for cell–cell communication through the known ligand‒receptor database. Interaction pairs whose ligands/receptors belong to the CD, VEGF, TNF, TGF, FGF, CCL, or CXCL families and have *P* values < 0.05 were used to evaluate the interactions between the cell populations.

### Correlation to bulk-RNA seq from clinical cohort

CibersortX and the average expression of signature genes were used to decipher the infiltration score of each cell subtype in the clinical cohort from TARGET. Then, the patients were divided into a high infiltration group and a low infiltration group for each cell cluster. The prognostic value of these clusters was assessed by Cox regression analysis. Spearman correlation analysis was performed to decipher the correlation between cell clusters (*P* values < 0.05 were considered meaningful correlations).

## Supplementary information

supplemental figures

Supplementary tables

## Data Availability

The datasets and supplemental information can be acquired online or by contacting the corresponding authors.
