# Supplementary material for: Characterizing the tumor microenvironment at the single-cell level reveals a novel immune evasion mechanism in osteosarcoma
Source: Bone Res. 2023 Jan 3;11:4. doi: 10.1038/s41413-022-00237-6 (PMC9810605; doi:10.1038/s41413-022-00237-6)
Supplement: Supplementary file 1 — supplemental figures [file 41413_2022_237_MOESM1_ESM.pdf]

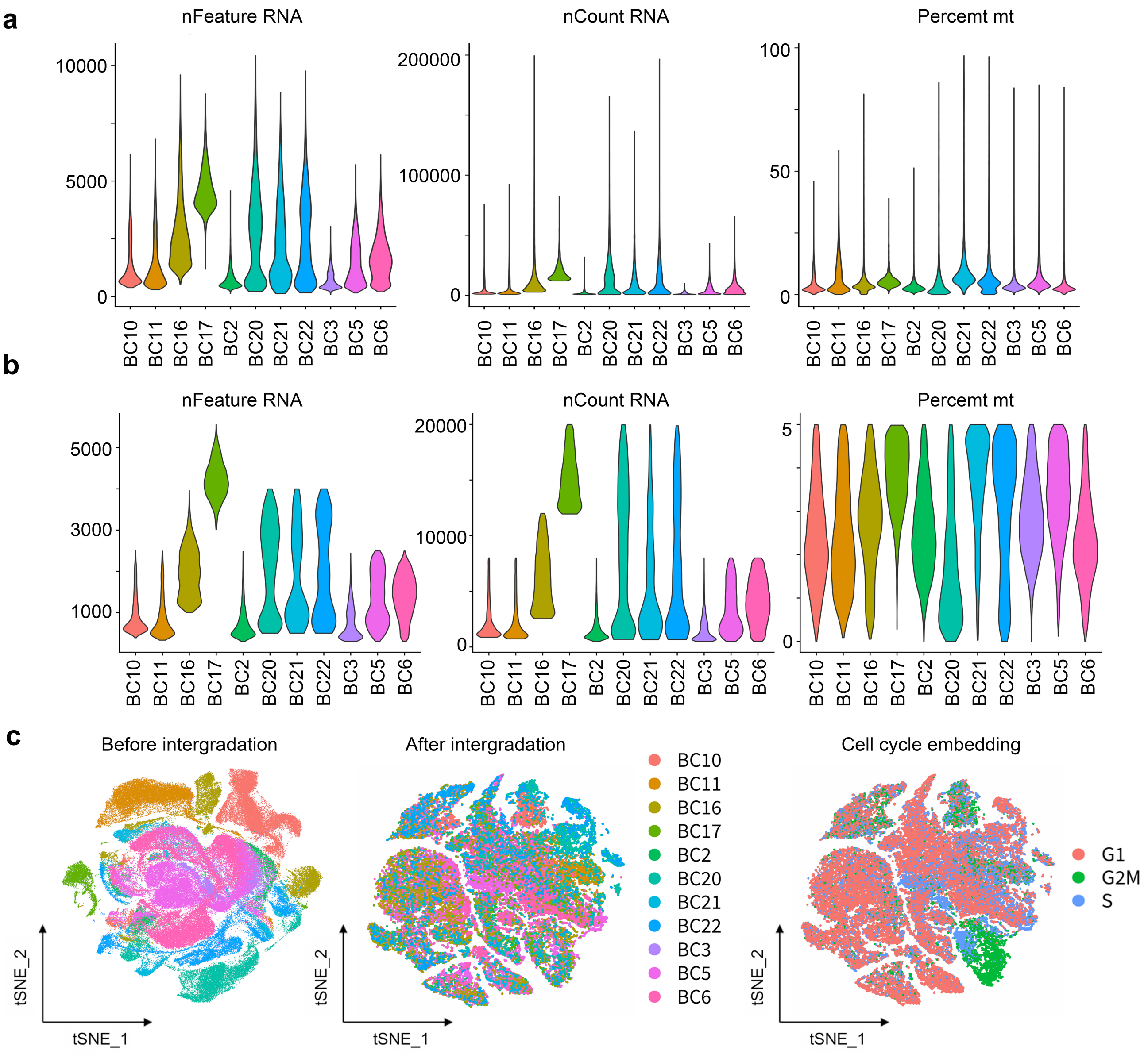
a
nFeature RNA
nCount RNA
Percemt mt
b
nFeature RNA
nCount RNA
Percemt mt
BC10
BC11
BC16
BC17
BC2
BC20
BC21
BC22
BC3
BC5
BC6
c
Before intergradation
After intergradation
Cell cycle embedding
G1
G2M
S
tSNE_1
tSNE_2

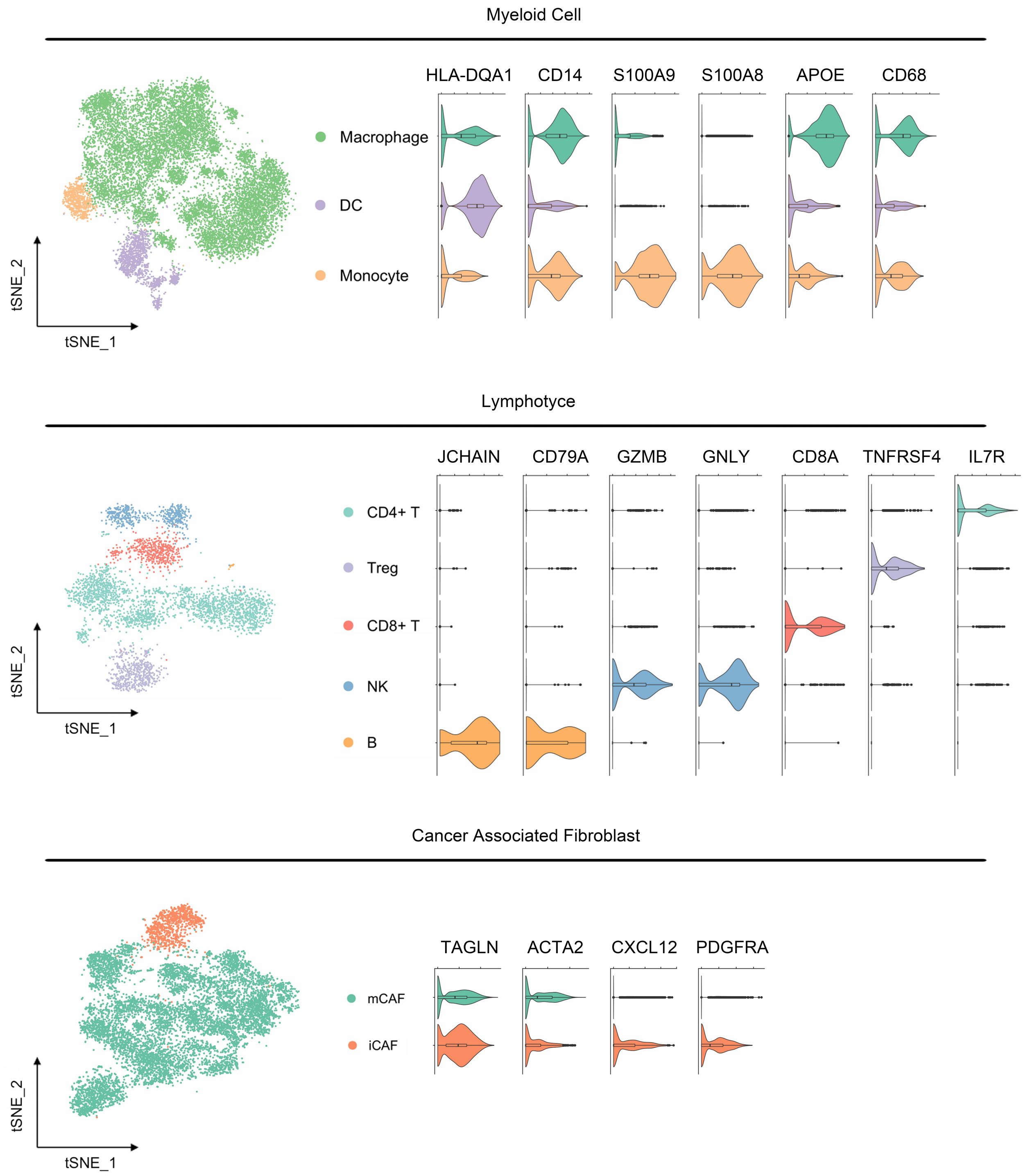

Myeloid Cell
HLA-DQA1
CD14
S100A9
S100A8
APOE
CD68
Macrophage
DC
Monocyte
tSNE_2
tSNE_1
Lymphotyce
JCHAIN
CD79A
GZMB
GNLY
CD8A
TNFRSF4
IL7R
CD4+ T
Treg
CD8+ T
NK
B
tSNE_2
tSNE_1
Cancer Associated Fibroblast
TAGLN
ACTA2
CXCL12
PDGFRA
mCAF
iCAF
tSNE_2
tSNE_1

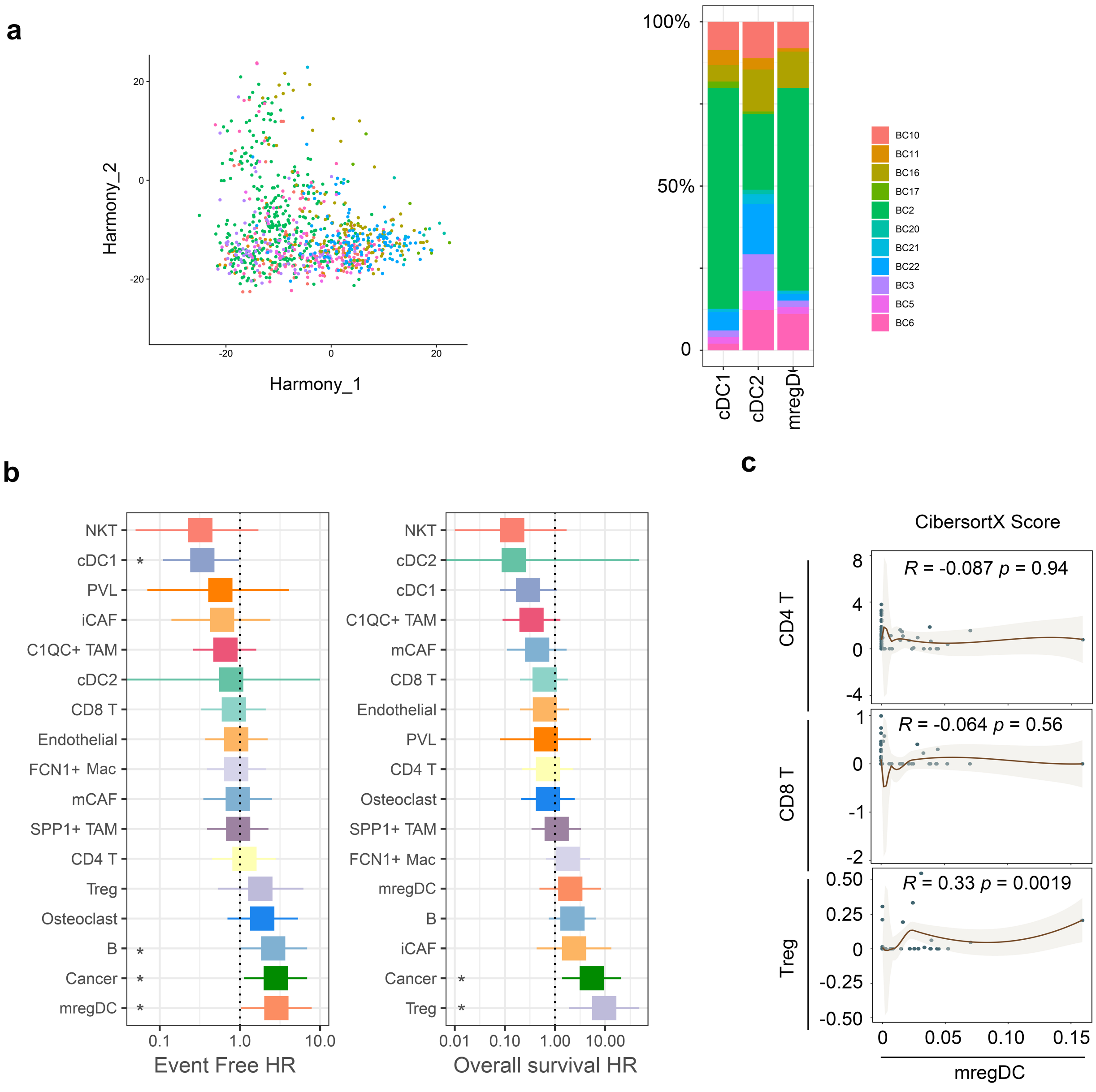
a
Harmony_2
Harmony_1
100%
50%
0
cDC1
cDC2
mregDC
BC10
BC11
BC16
BC17
BC2
BC20
BC21
BC22
BC3
BC5
BC6
b
NKT
cDC1
PVL
iCAF
C1QC+ TAM
cDC2
CD8 T
Endothelial
FCN1+ Mac
mCAF
SPP1+ TAM
CD4 T
Treg
Osteoclast
B
Cancer
mregDC
0.1
1.0
10.0
Event Free HR
0.01
0.10
1.00
10.00
Overall survival HR
c
CibersortX Score
R = -0.087 p = 0.94
R = -0.064 p = 0.56
R = 0.33 p = 0.0019
CD4 T
CD8 T
Treg
mregDC

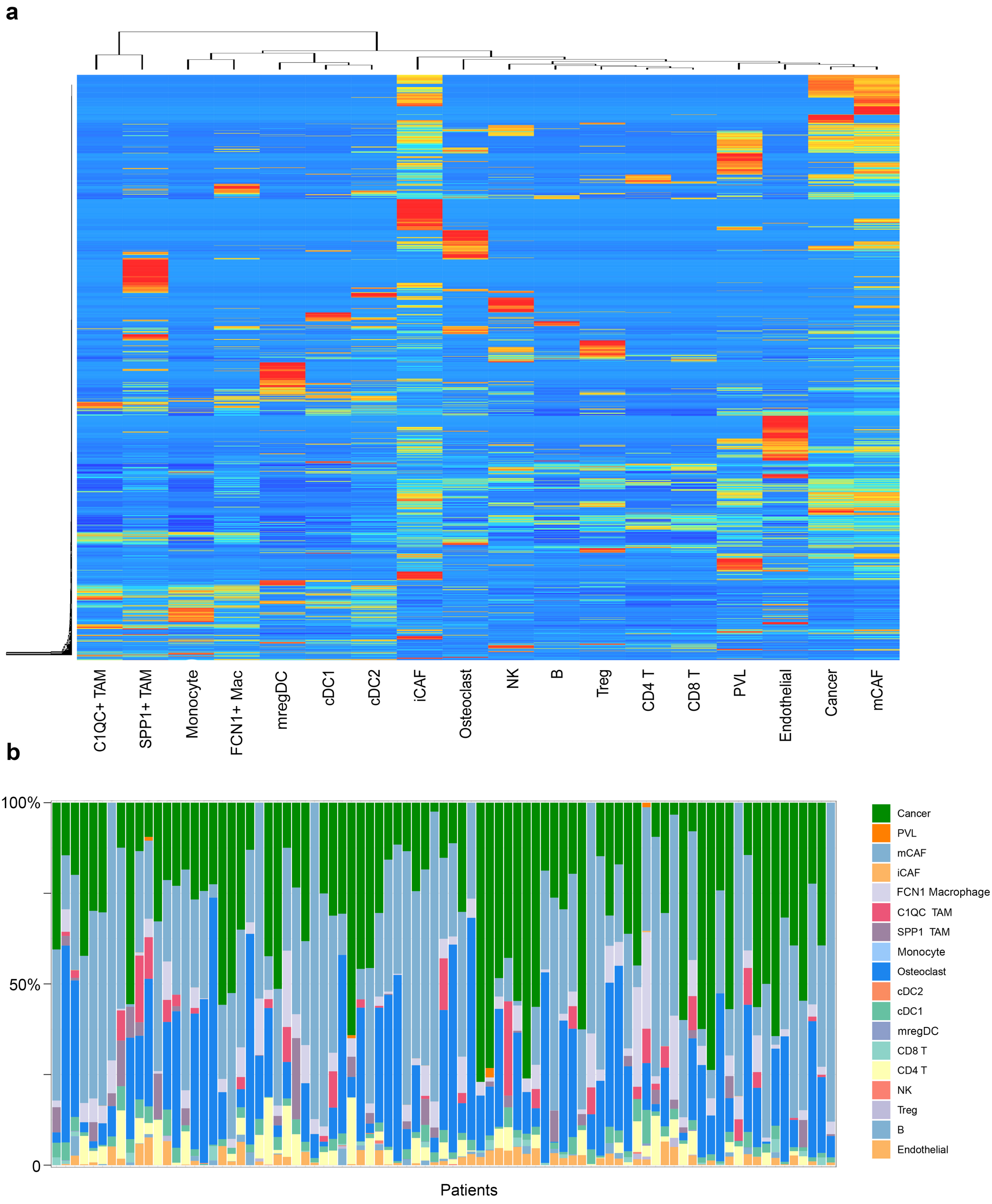
a
C1QC+ TAM
SPP1+ TAM
Monocyte
FCN1+ Mac
mregDC
cDC1
cDC2
iCAF
Osteoclast
NK
B
Treg
CD4 T
CD8 T
PVL
Endothelial
Cancer
mCAF
b
100%
50%
0
Patients
Cancer
PVL
mCAF
iCAF
FCN1 Macrophage
C1QC TAM
SPP1 TAM
Monocyte
Osteoclast
cDC2
cDC1
mregDC
CD8 T
CD4 T
NK
Treg
B
Endothelial

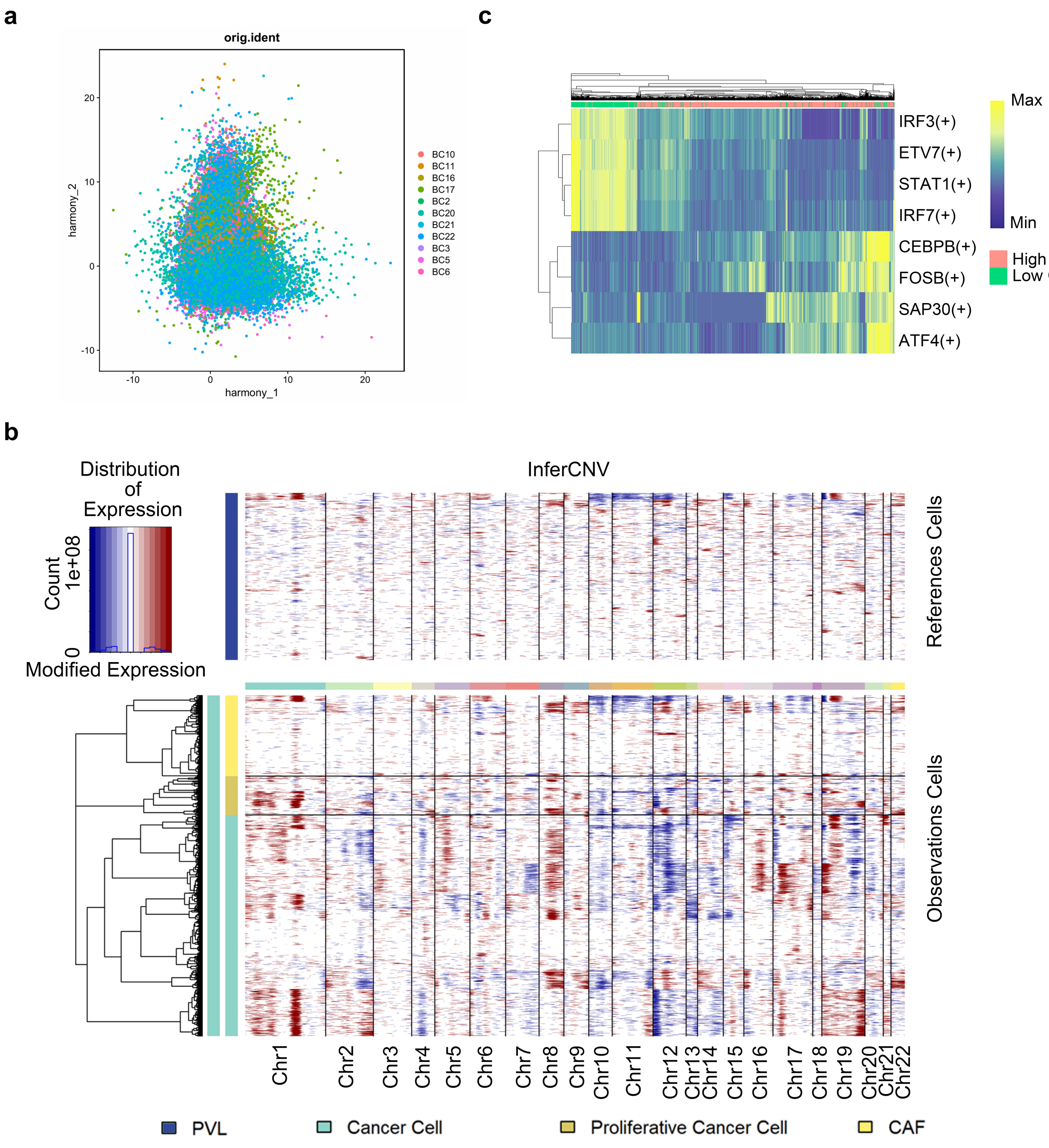
a
orig.ident
harmony_2
harmony_1
BC10
BC11
BC16
BC17
BC2
BC20
BC21
BC22
BC3
BC5
BC6
c
IRF3(+)
ETV7(+)
STAT1(+)
IRF7(+)
CEBPB(+)
FOSB(+)
SAP30(+)
ATF4(+)
Max
Min
b
Distribution of Expression
Count 1e+08
0
Modified Expression
InferCNV
References Cells
Observations Cells
Chr1
Chr2
Chr3
Chr4
Chr5
Chr6
Chr7
Chr8
Chr9
Chr10
Chr11
Chr12
Chr13
Chr14
Chr15
Chr16
Chr17
Chr18
Chr19
Chr20
Chr21
Chr22
PVL
Cancer Cell
Proliferative Cancer Cell
CAF

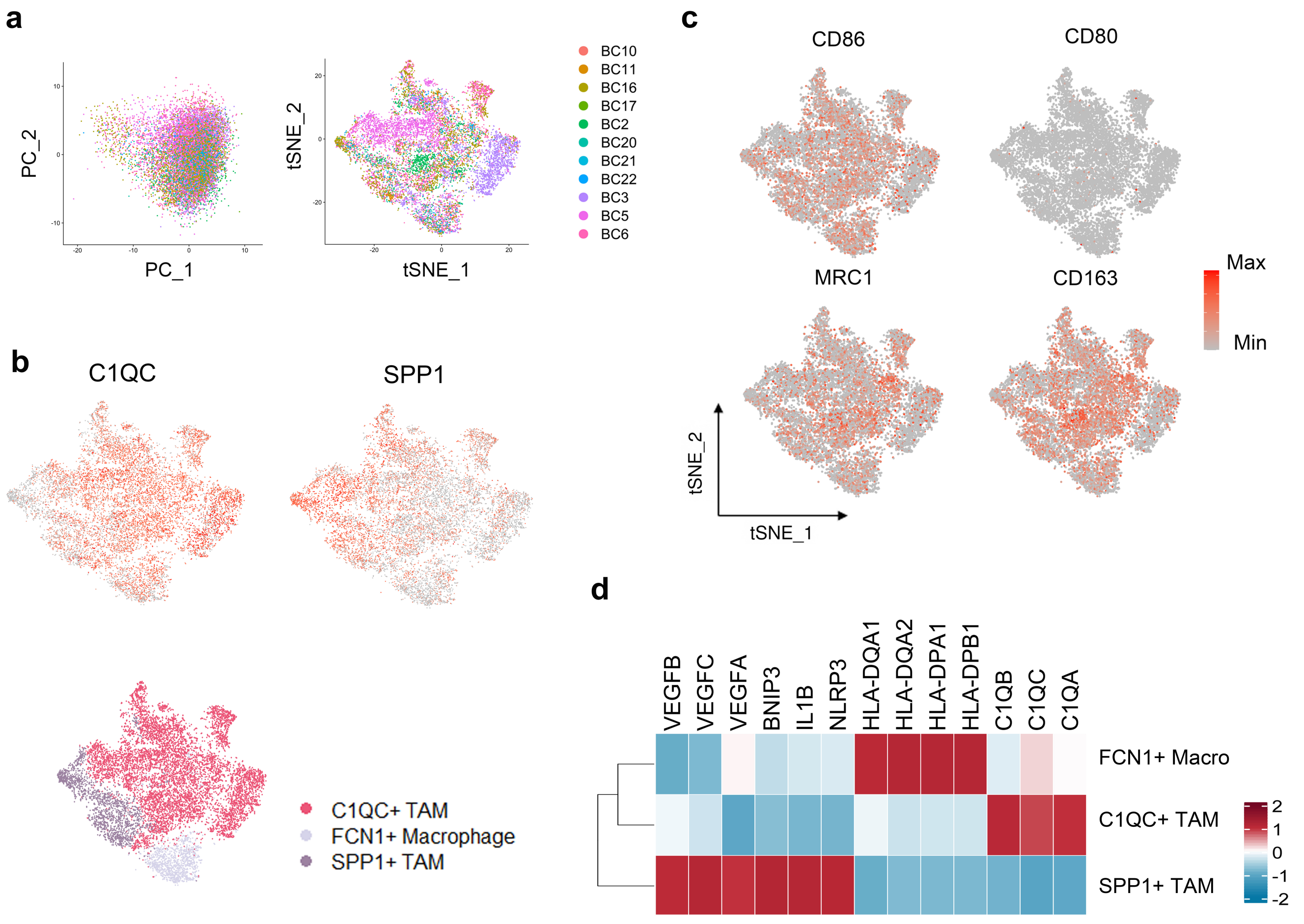

a
PC_2
PC_1
tSNE_2
tSNE_1
BC10
BC11
BC16
BC17
BC2
BC20
BC21
BC22
BC3
BC5
BC6
b
C1QC
SPP1
C1QC+ TAM
FCN1+ Macrophage
SPP1+ TAM
c
CD86
CD80
MRC1
CD163
Max
Min
tSNE_2
tSNE_1
d
VEGFB
VEGFC
VEGFA
BNIP3
IL1B
NLRP3
HLA-DQA1
HLA-DQA2
HLA-DPA1
HLA-DPB1
C1QB
C1QC
C1QA
FCN1+ Macro
C1QC+ TAM
SPP1+ TAM
2
1
0
-1
-2

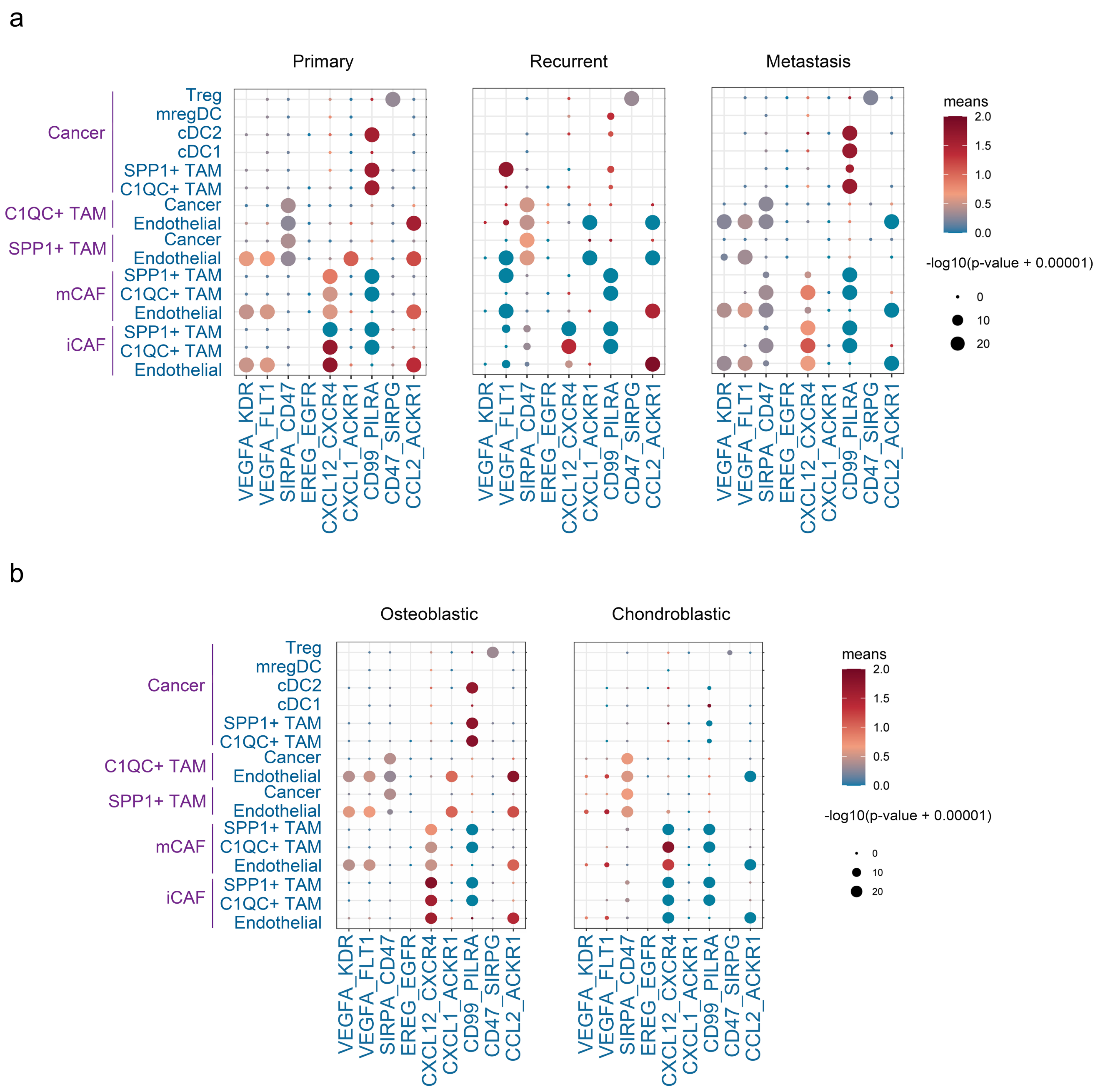

a
Primary
Recurrent
Metastasis
Cancer
C1QC+ TAM
SPP1+ TAM
mCAF
iCAF
Treg
mregDC
cDC2
cDC1
SPP1+ TAM
C1QC+ TAM
Cancer
Endothelial
VEGFA_KDR
VEGFA_FLT1
SIRPA_CD47
EREG_EGFR
CXCL12_CXCR4
CXCL1_ACKR1
CD99_PILRA
CD47_SIRPG
CCL2_ACKR1
means
2.0
1.5
1.0
0.5
0.0
-log10(p-value + 0.00001)
0
10
20
b
Osteoblastic
Chondroblastic

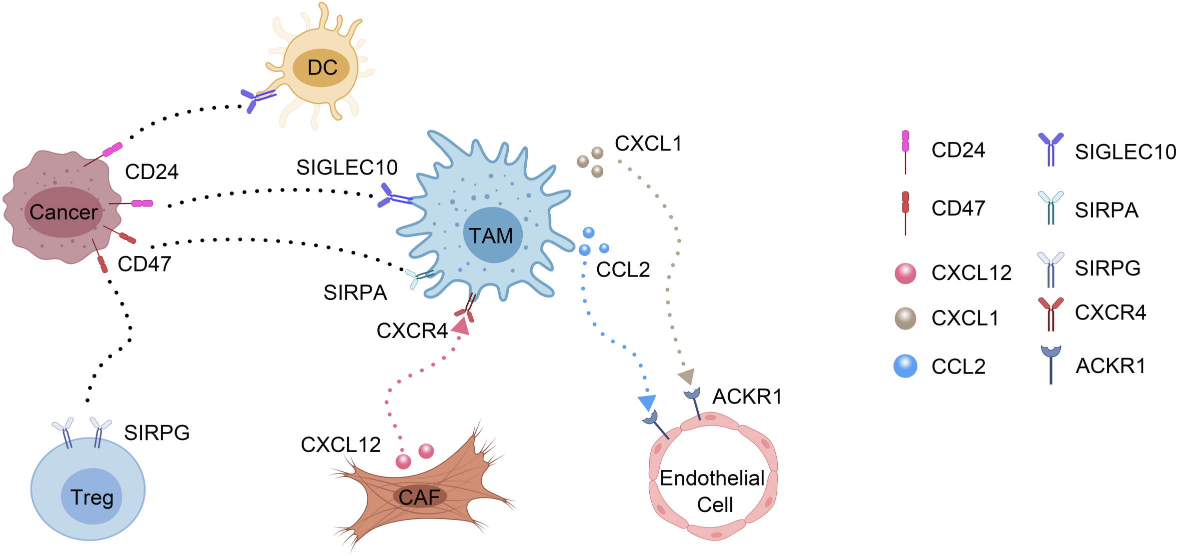

DC
CD24
Cancer
SIGLEC10
CXCL1
TAM
CCL2
CD47
SIRPA
CXCR4
SIRPG
Treg
CXCL12
CAF
ACKR1
Endothelial Cell
CD24
CD47
CXCL12
CXCL1
CCL2
SIGLEC10
SIRPA
SIRPG
CXCR4
ACKR1
