# Supplementary material for: Characterizing the tumor microenvironment at the single-cell level reveals a novel immune evasion mechanism in osteosarcoma
Source: Bone Res. 2023 Jan 3;11:4. doi: 10.1038/s41413-022-00237-6 (PMC9810605; doi:10.1038/s41413-022-00237-6)
Supplement: Supplementary file 2 — Supplementary tables [file 41413_2022_237_MOESM2_ESM.docx]

Table S1 Sequence of probe and siRNA used in the paper

| CD24-H probe | TTGTTGTTTCACTGGAATAAATCTGCGTGGGTAGGAGC |
| --- | --- |
| siCd24a #1 sense | CCAAACAUCUGUUGCACCGUUTT |
| siCd24a #1 antisense | AACGGUGCAACAGAUGUUUGGTT |
| siCd24a #2 sense | CAAAUCCAAGUAACGCUACCATT |
| siCd24a #2 antisense | UGGUAGCGUUACUUGGAUUUGTT |
| siNC sense | UUCUCCGAACGUGUCACGUTT |
| siNC antisense | ACGUGACACGUUCGGAGAATT |

Table S2 Sequence of primers used in the paper

| Gapdh F | AGCAAGGACACTGAGCAAGA |
| --- | --- |
| Gapdh R | GGGGTCTGGGATGGAAATTGT |
| Cd24a F | CATCTGTTGCACCGTTTCCC |
| Cd24a R | GAGACGTTTCCTGGCCTGAG |
